# The Immediate Effects of Immersive Virtual Reality on Autonomic Nervous System Function in Patients with Disorders of Consciousness after Severe Acquired Brain Injury: A Pilot Study

**DOI:** 10.3390/jcm12247639

**Published:** 2023-12-12

**Authors:** Giuseppe Reale, Augusto Fusco, Rossella Calciano, Noemi Vallario, Gabriele Vagnarelli, Pietro Caliandro, Letizia Castelli, Marco Moci, Gaetano Tieri, Luigi Iasevoli, Luca Padua

**Affiliations:** 1 UOC Neuroriabilitazione ad Alta Intensità, Fondazione Policlinico Universitario A. Gemelli IRCCS, Largo Agostino Gemelli 8, 00168 Rome, Italyaugusto.fusco@policlinicogemelli.it (A.F.); marco.moci@gmail.com (M.M.);; 2 Department of Geriatrics and Orthopaedics, Università Cattolica del Sacro Cuore, 00168 Rome, Italy; 3 UOC Neurologia, Fondazione Policlinico Universitario A. Gemelli IRCCS, 00168 Rome, Italy; 4 Virtual Reality and Digital Neuroscience Lab, Department of Law and Digital Society, University of Rome Unitelma Sapienza, Piazza Sassari, 4, 00161 Rome, Italy; gaetano.tieri@unitelmasapienza.it; 5 IRCCS Fondazione Santa Lucia, 00179 Rome, Italy; 6 Multiple Sclerosis Unit, IRCCS Fondazione Santa Lucia, 00179 Rome, Italy

**Keywords:** skin conductance response, immersive virtual reality, disorder of consciousness, autonomic nervous system, electrodermal activity

## Abstract

Disorders of Consciousness (DoCs) after severe acquired brain injury involve substantial impairment of cognition and physical functioning, requiring comprehensive rehabilitation and support. Technological interventions, such as immersive Virtual Reality (VR), have shown promising results in promoting neural activity and enhancing cognitive and motor recovery. VR can induce physical sensations that may activate the Autonomic Nervous System (ANS) and induce ANS-regulated responses. This study aimed to investigate the effects of immersive VR on the ANS in patients with DoCs through the analysis of the electrodermal activity (EDA). EDA was measured with a wearable device during a single immersive VR session consisting of static and dynamic videos depicting naturalistic environments. A pilot case–control study was conducted with 12 healthy participants and 12 individuals with DoCs. Results showed higher EDA values in patients than in healthy participants (*p* = 0.035), suggesting stronger autonomic activation during immersive VR exposure, while healthy subjects, in turn, showed a decrease in EDA values. Our results revealed a significant interaction between conditions and groups (*p* = 0.003), with patients showing significantly increased EDA values from the baseline compared to dynamic video observation (*p* = 0.014) and final rest (*p* = 0.007). These results suggest that immersive VR can elicit sympathetic arousal in patients with DoCs. This study highlights the potential of immersive VR as a tool to strengthen autonomic responses in patients with impaired consciousness.

## 1. Introduction

Disorder of consciousness (DoC) refers to a state of prolonged altered consciousness due a severe acquired brain injury (sABI), ranging from coma to a minimally conscious state based on neurobehavioral and motor function [1,2]. Various cognitive and physical functions may be impaired, affecting the ability to communicate, think, move, and perform daily activities [3].

In recent years, the integration of rehabilitation with technology has offered innovative tools to promote neural activity and improve recovery. Many new therapeutic approaches, such as robotic therapy, electrical or magnetic brain stimulation, and invasive and non-invasive vagus nerve stimulation, have been proposed for restorative or adaptive purposes, either as add-on, online, or stand-alone interventions [4,5,6,7]. Additionally, immersive virtual reality (VR) has shown promising results for treating people with impaired cognitive functions. Individuals with sABI can benefit from interactive VR experiences tailored to their specific impairments [8]. Through the stimulation of multisensory channels, VR rehabilitation may promote long-term effects on brain excitability and neuronal plasticity [9,10].

VR develops computer-mediated environments, enhancing interactions similar to real life. Through immersive VR, the perception of the real world is replaced by the perception of the virtual world, transferring (or reinforcing) the relative consciousness of the real world [11]. The user can experience a panoramic image or 360° video as if they were inside the environment; by moving their head, the individual’s position adapts to the virtual landscape, giving a sense of presence in the environment [12,13,14]. Different sensorial elements (visual, auditory, haptic, vestibular, proprioceptive, and finally, olfactory and gustatory) can be integrated to achieve and/or enhance the aimed outcomes in several diseases [15,16]. The intensity of the responses to virtual stimuli depends on the alignment of the sensory and motor virtual stimuli with the real world [15,17].

Rehabilitation of patients with DoCs requires a combination of personalized interventions, targeting stimuli in order to regain consciousness. A customized stimulus (such as music) can evoke emotions, influencing the Autonomic Nervous System (ANS) and the neuroendocrine system [18,19], as well as improve mental performance for different cognitive skills, including arousal [20]. VR can induce physical sensations that may activate the ANS and induce ANS-regulated responses [21]. Brain responses underlying perceptual dimensions have been found to be related to motor system activation through embodied mechanisms, which include the simulation of actions, emotions, and bodily sensations [22]. Moreover, the individualized preferences of patients with DoCs may be difficult to comprehend in clinical practice, imposing the therapeutic choices towards general stimuli or information given by the caregiver. The presence of a response to a particular stimulus could provide a functional method to examine the sense of awareness among patients with DoCs in an objective manner. By evaluating this brain activity, additional information can be also gathered to improve the clinical examination and functional rehabilitation [23].

Few studies have investigated how VR may stimulate the ANS and which changes can be recorded in patients with DoCs. In this study, we examined whether and how immersive VR influenced ANS function in patients with DoCs after sABI. Specifically, we assessed the changes in electrodermal activity (EDA) induced by static and dynamic 360° videos during a single exposure through a VR headset. EDA has been shown to be a valuable tool for studying the emotional response after musical or odor stimulation [24]. The analysis of ANS responses could be valuable in patients experiencing DoCs as it permits the identification of distinct neural patterns associated with awareness, particularly when behavioral responses are not evident [25].

The distinction between static and dynamic videos in immersive VR was aimed at differentiating which subjects may be more activated in the vegetative system in response to different kinematic stimuli [26].

## 2. Materials and Methods

### 2.1. Study Design and Assessment

This was a pilot, case–control, interventional study.

Two groups (Healthy Group, HG; Patient Group, PG) underwent a single 25 min immersive VR session of successive and continuous phases using Oculus Go^®^, structured as follows: an initial 5 min phase (without images), T0 (baseline); a 5 min static video, T1; a 5 min wash-out phase (without video), T2 (rest); a 5 min dynamic video, T3; and a final 5 min phase, T4 (rest). Landscape environments characterized the static videos. In the dynamic videos, changes in landscape were depicted in the same type of environment (naturalistic backgrounds), thus creating a sense of movement and progression within the virtual environment. It has been shown that the natural environment produces relaxation effects and improves physical health in healthy individuals [27,28], with similar effects being replicated from an analogous scenario in immersive VR [29,30].

In order to eliminate stressful or distracting conditions derived from the hospital setting, both groups were tested supine, during the afternoon, in the same quiet room of our ward [11]. No interaction with researchers was allowed during the experimental session, even though a physician was always present due to clinical safety reasons. Healthy individuals were instructed to behave as usual without receiving any specific instructions. All enrolled subjects received the same materials, with all hygienic procedures observed.

To assess the effects of immersive VR on the ANS, we recorded EDA, which is considered a non-invasive, real-time measure of ANS [31]. EDA is linked to the activity of the sweat glands on the skin, controlled by the ANS. During the session, we measured changes in EDA by placing an E4^®^ wristband on the subjects’ right wrists (Empatica Inc., Cambridge, MA, USA). This non-invasive wearable device is equipped with two stainless steel sensors placed on the volar surface of the wrist, which allow continuous recording of the skin’s electrical conductance. EDA measurements were recorded at a sampling rate of 4 Hz, with a sensitivity range of 0.01–100 µS. For each time interval, the wristband recorded EDA values for the duration of the experimental trial; the mean value for each stage was calculated and used in the subsequent statistical analysis. At the beginning and at the end of each phase, the experimenter recorded the EDA values to ensure time alignment between the physiological recordings and the phases. During all experimental sessions, the evaluation quantified EDA levels.

The research was conducted in accordance with the International Guidelines for Good Clinical Practice and The Declaration of Helsinki and received approval from the Institutional Ethics Committee (n. 0038186/21). Prior to participation, all individuals or their legal guardians provided written informed consent.

### 2.2. Participants

Twenty-four individuals were included in the study, including twelve healthy adults (HG) and twelve patients with a DoC resulting from an sABI (PG). We enrolled patients during their rehabilitative course, in stable clinical conditions, out of uncontrolled epilepsy or neuro-vegetative crisis.

Inclusion criteria for PG were age between 18 and 90 years, experiencing an acquired traumatic or non-traumatic brain injury in the previous six months, with a disorder of consciousness classified as a minimally conscious state (MCS) or unresponsive wakefulness syndrome (UWS) according to the scores of the Coma Recovery Scale-revised (CRS-r) with scores of the Level of Cognitive Functioning (LCF) scale ≤ 3. We also evaluated the Glasgow Coma Scale at the enrollment of the study.

The CRS-R is a standardized neurobehavioral assessment measure for individuals with DoCs, furnishing a clear diagnosis, monitoring behavioral recovery, predicting outcomes, and assessing treatment effectiveness [32]. The LCF scale is an early development used to assess cognitive function for planning treatment, monitoring recovery, and categorizing outcomes in patients after coma [33]. The GCS describes impaired consciousness according to different grades of responsiveness (eye, motor, and verbal responses), thus providing an easy and clear way to describe patients with DoCs [34].

Exclusion criteria were severe pre-existing or auditory or visual impairments (in the history of the patient as obtained by the caregiver or after the brain injury as revealed by evoked potentials and referred specialist visits, routinely performed at the admission to our ward), and the concomitant use of drugs known to affect the ANS response.

### 2.3. Virtual Reality Intervention

**Figure 1:**
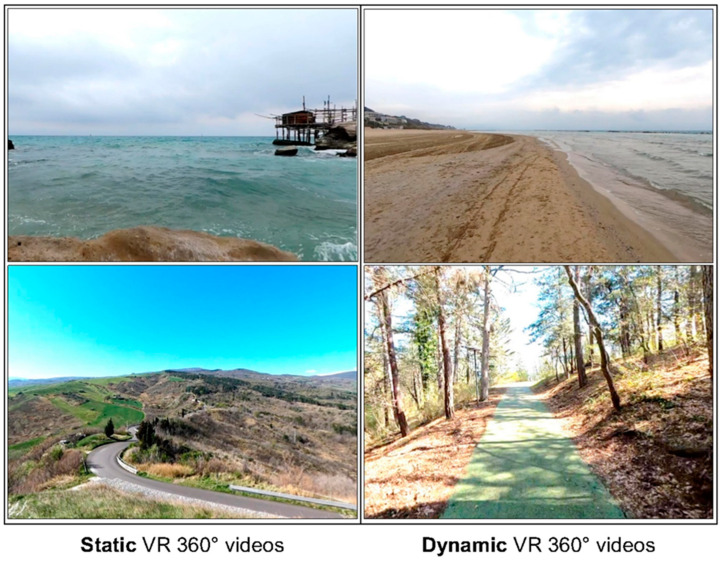
Examples of 360° VR videos of naturalistic environments presented through the Oculus Go Head-Mounted display. (**Left**) snapshots of static videos (without any camera movement); (**right**) snapshots of dynamic videos (with camera movement similar to a human walking forward).

Immersive virtual experiences have been created by means of 360° VR videos that reproduce naturalistic scenarios in static and dynamic versions. Immersion in natural environments (virtual or real) has been shown to have physiological and psychological effects on relaxation and well-being [35,36,37]. The virtual reality videos were taken with a GoPro Max^®^ 360° camera (GoPro Inc., San Mateo, CA, USA), with a spherical resolution of 5376 × 2688 at 30 fps and 3D spatial audio with an external Zoom H1 microphone. We recorded two different naturalistic seaside and mountain locations in known places (Abruzzo, Italy), both with a static point of view (camera recording the environment in a fixed position) and with a dynamic point of view (the cameraman walked slowly with the camera pointed in front of him). The spherical 360° videos were processed with Adobe Premier Pro^®^ 2021 (Version 15) (Adobe, San Jose, CA, USA) and installed on Oculus Go^®^ (Meta, Menlo Park CA, USA), with a resolution of 1.440 by 1.280 (per eye), a refresh rate of 72 Hz, and 3 degrees of freedom (DoF). Figure 1 shows a frame of the videos in both the dynamic and static conditions.

Participants wore a head-mounted display (HMD) for the presentation of stereoscopic images and three-dimensional sounds, allowing the user to look around in the virtual environment. The experimental trial setting was set up to facilitate the virtual experience with their visual and auditory senses heavily immersed. Participants observed the immersive 360° video only in static and dynamic video conditions, while in the other conditions (rest), no videos were displayed within the HMD.

### 2.4. Data Analysis

The age and sex of the two groups were analyzed using *t*-tests. We extracted the mean value of the 5 min window, consisting of the exposure time of each condition, which included baseline, observation of static video, rest, observation of dynamic video, and final rest. Data were then normalized with log10 transformation and analyzed with a parametric repeated measures ANOVA with conditions (baseline, static video observation, rest, dynamic video observation, final rest) as within factors and group (patient or healthy group) as between factors. Analogously, the same statistics were performed for the analysis between subgroups in PG. Multiple comparison tests were performed post hoc using the Newman–Keuls test.

## 3. Results

The study sample consisted of 24 individuals, divided into two groups: HG, consisting of 12 healthy adults (five females, seven males; mean ± SD, 28.1 ± 6.8 years), and PG, consisting of 12 patients with a DoC resulting from an sABI (four females, eight males; mean ± SD, 45.6 ± 23.7 years). Table 1 shows the clinical characteristics at the baseline of sABI patients with DoCs, while Table 2 reports the detailed scores of CRS-r for all the patients.

**Figure 2:**
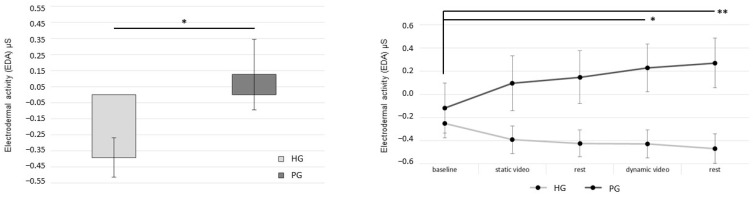
Electrodermal activity (EDA) recorded across experimental conditions for both healthy and patient group. (**Left**) graph represents the mean value of EDA between healthy group (HG) and patient group (PG). (**Right**) graph represents the significant interaction between conditions × groups with higher EDA values for patients vs. healthy group across conditions. ** = *p* < 0.01; * = *p* < 0.05.

The two groups were significantly different in terms of age (*p* = 0.044) but not in terms of sex. At the baseline, there was no difference in EDA values between groups (*p* = 0.59). Repeated measures ANOVA revealed a significant effect between groups (F(1, 22) = 5.04, *p* = 0.035), with high EDA values in the PG (means ± SD; 0.12 ± 0.76) compared to low values in the HG (means ± SD; −0.4 ± 0.42), suggesting more robust autonomic activation in patients after VR exposure. Statistical analyses revealed no significant effect of conditions (F(4, 88) = 0.41, *p* = 0.80). A significant interaction between conditions x groups was found (F(4, 88) = 4.31, *p* = 0.003), accounting for higher EDA values in the PG than in the HG. The post hoc analysis showed a significant difference between baseline compared to dynamic video observation (*p* = 0.014) and final rest (*p* = 0.007), and a tendency close to the significance between values at baseline and static video observation (*p* = 0.06) and rest (*p* = 0.053) for the PG only. In contrast, no differences were found for the HG between conditions (Figure 2).

These results suggest that while healthy participants were not physically activated by virtual exposure, showing a decrease in EDA activity in all conditions, the patients were significantly stimulated by VR videos. The EDA activity increased over time across all conditions.

**Figure 3:**
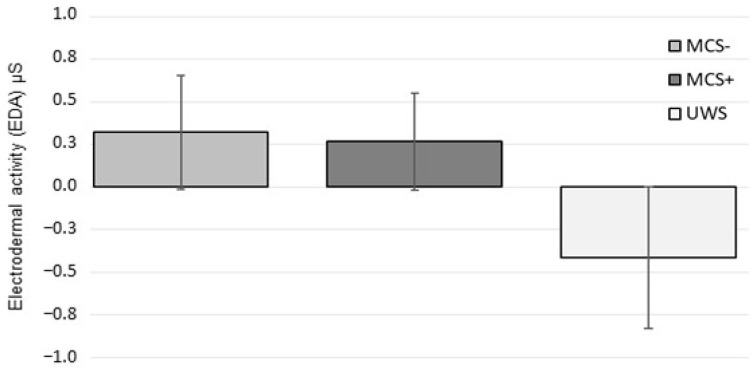
Level of EDA for the different subgroups (Unresponsive Wakefulness Syndrome, UWS; Minimally Conscious State, MCS− and MCS+) in PG. Means ± SD: UWS −0.41 ± 0.72; MCS− 0.32 ± 0.78; MCS+ 0.27 ± 0.74.

No significant differences in PG between DoC subgroups (UWS/MCS−/MCS+; *p* = 0.337) and no significant interactions between subgroups and conditions (*p* = 0.136) were found (Figure 3).

**Figure 4:**
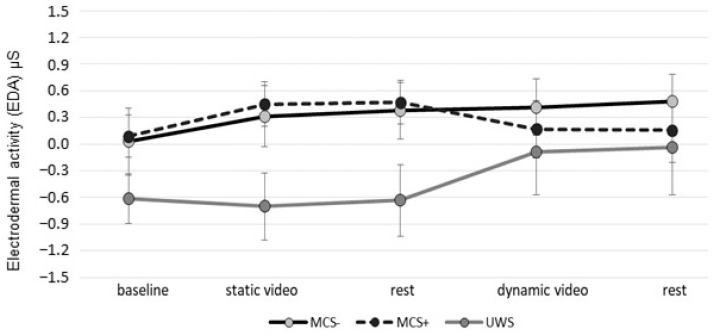
The EDA data for the different subgroups in the different phases of the experimental trial, with a different trend noted between MCS+ and UWS. No significant differences between groups were found.

Interestingly, we report a different trend of EDA levels in MCS+, tending to decrease, and UWS, tending to increase, in the final phases (dynamic video and final rest) (Figure 4).

## 4. Discussion

Our study supports the hypothesis that immersive VR may elicit an activation of the ANS, in particular with an increased arousal of the sympathetic nervous system. We found significantly higher EDA values in the PG during the immersive VR session than in the HG. Activation of the ANS appears from the first phase of immersive VR activity (static video) and continues in all subsequent phases of immersive VR activity (rest phase–dynamic video–terminal rest phase) with a gradual increase in absolute EDA values (even with large SDs). Patients showed significant differences between the baseline and dynamic video and between the baseline and terminal resting phase. No differences were found between the intermediate phases, even if with thresholds very close to statistical significance compared with the baseline. No differences between subgroups were found in the PG. In healthy subjects, no significant differences were found within the group and for the different experimental phases.

We are unable to speculate on the reason why this variation in physiological values occurs in the PG, nor its significance. Immersive VR induced a progressive activation, independent of the scenario (dynamic vs. static). Higher EDA signals in the PG (sympathetic stimulation) could be related to positive affective arousal (emotional response) or negative affective arousal (overstimulation, cybersickness, or generic fear), based on individual perception [38]. The use of known content (naturalistic scenario) could have enhanced the response of the ANS detected with EDA as possible emotional meaning in patients, although the novelty of the type of intervention may have concurred. Emotional processes determined by activation of ANS have also been shown with preferred music [39]. At the moment, we do not have realistic evidence of emotion as the main factor of activation revealed by EDA.

The creation of realistic environments in immersive VR can evoke strong physiological adaptations, determined by an increased sense of presence. Visual feedback during virtual reality is able to activate a network of cortical regions, including the superior and inferior parietal cortex, ventral premotor cortex, primary motor cortex, dorsal premotor cortex, inferior frontal gyrus, and supplementary motor areas [40,41,42]. In particular, the frontal and orbitofrontal areas and the left temporal region are the brain areas most involved in the sense of presence and in visuospatial exploration of more familiar and pleasant environments, based on analysis of EEG data [42]. On the contrary, it has also been described that experiences with certain stimuli within a natural environment can also negatively influence physiological parameters [43].

We found a different ANS activation between PG and HG. This could be determined by the different meanings interpreted by the levels of consciousness. In this sense, we noted a different trend of EDA values of UWS (progressive activation) and MCS+ (progressive reduction after initial activation). Due to the scarcity of enrolled patients in the different subgroups (three UWS; six MCS−; three MCS+), we are unable to draw clear conclusions. Currently, too many conditions may have determined the observed changes (devices, content of intervention, personal experience in patients’ lives before the accident, etc.). All these factors must be carefully assessed in future studies.

In HG, we found a progressive decrease in EDA signals as a possible relaxing effect. These results are in line with those obtained by Gerber and colleagues [43], in which a restorative effect on the physiological and psychological state of healthy enrolled participants was demonstrated. In another study, a VR intervention with naturalistic scenes had a relaxing effect on healthy subjects in an experimental setting similar to ours, using head-mounted displays while lying in bed, with a reduction in heart rate variability and improved visual processing [44]. This physiological activation is also demonstrated by the use of stress-related visual images, which have been shown to have a significant impact on posture, resulting in an increase in stress-related factors [45]. In our experiment, we generated dynamic, non-stressful scenarios set in a naturalistic context with movement. It is possible that the sounds of the natural environment also contributed to our findings [11,46].

With regard to PG, the results of our study contrast with those of Luauté and colleagues [24]. They found that the skin conductance of healthy subjects increased when exposed to preferred music and odors. Researchers suggested that the electrodermal responses of healthy individuals could be attributed to the intensity of emotions evoked by specific stimuli. However, no significant difference was observed among patients with DoCs. They suggested that the inability to elicit electrodermal responses in patients could be related to differences in the analysis of electrodermal activity levels (tonic and phasic signals) with regard to the given stimulus. According to our hypothesis, the variation in outcomes can be attributed mainly to the type of intervention used, differentiating between music/odors and immersive VR.

For both groups, no negative effects from immersive VR were recorded during the experimental session or afterward. More discomfort was found in a digital urban environment due to unexpected turns [43]. In our study, there were no turns during the movement into the virtual natural tour. The PG was not able to explore the surrounding space due to their severe clinical conditions. VR-based rehabilitation can cause dizziness, nausea, eye fatigue, and gait instability due to sensory conflict between the virtual and physical environments [47]. Prolonged exposure to VR can also lead to fatigue and discomfort, resulting in unpleasant feelings such as cyber/mobility sickness [48,49]. For this condition, we selected a brief time of exposure to virtual stimuli (5 min). Several authors have argued that virtual environments should be designed to minimize potential negative side effects, both immediate and long-term [50,51].

Our findings have to be considered in light of some limitations. The main limitation is related to the small sample size. In a similar study, an analogous sample size was enrolled [24]. We used a cross-sectional design for our experimental trial. In this topic, studies are still very few and varied, with sample sizes being too small and statistical power and diversity in the outcomes obtained being insufficient to ascertain a clear effect. Furthermore, our HG was significantly younger (young adults) than the PG, similar to the sample recruited in an analogous study (seven healthy subjects, four women and three men, aged from 21 to 44 years with an average age of 28.7 years) [24]. All participants were analyzed without any information regarding their previous experiences or differences in culture, lifestyle, and education. It has been shown that cognitive reserve can help to address patients toward technological or conventional rehabilitation [52,53]. A more in-depth analysis of patients’ physical and mental activity history may help understand the skin conductance response.

Our results are partly inconsistent with previous findings concerning EDA levels or mechanisms of action on the ANS in DoC patients. In these types of patients, a potential limitation may also be related to participants’ clinical cognitive and motor conditions, which may influence the response to VR stimulation. Appropriate baseline measurement and normalization of the clinical requirements are necessary, varying significantly in sABI. Additionally, it remains unclear whether the alterations noted during the audiovisual intervention depend on the content or the type of VR. The integration of this information with other physiological measurements could help to identify the subgroup of DoC patients who are more likely to show autonomic responses to VR stimulation. The physiological values determined by the activation of ANS need to be gradually adapted during the VR in healthy individuals [54]. This could also be valid for individuals with DoCs. Thus, EDA signals could be analyzed over time during rehabilitation in order to determine a different course of action. Finally, we did not specifically evaluate the possible presence of dysautonomia, even if all the enrolled patients were enrolled in stable clinical conditions. It is possible that our results could be influenced by the activation of related phenomena and not only by the autonomic sympathetic arousal. All these possible influencing factors need to be assessed in future studies in order to confirm our results.

The customization of VR intervention depends on technological manipulations of content, sounds, and images. At present, we are not able to determine which elements would have a greater impact and whether or how they would interact to achieve outcomes, inducing a generic caution in its use. The degree of misalignment between the real and virtual environments directly impacts the level of immersion and embodiment [15,17]. The use of simultaneous audiovisual immersive scenariost enabled the participants to feel a more intensive experience of the virtual context, diminishing the possible distraction derived by the real world (ambient sounds, brightness, presence of other people in the physical environment) [11]. An effective method of discriminating between the auditory and visual content of immersive VR sessions is to use a validated questionnaire [55]. In individuals with DoCs, it is not possible to access this information due to patients’ clinical conditions. Additionally, we did not verify if the patients had their eyes open during the session. It could be useful to better evaluate the EDA signals in response to specific VR stimuli with an eye tracker, obtaining more data. This could be useful in developing specific technological components for better therapeutic outcomes or to reduce distressing elements. Furthermore, we did not experiment with augmented or mixed reality. It is essential to evaluate all these technologies in future studies to identify the potential role of augmented or mixed-nature immersive VR in determining autonomic and cognitive responses in patients with impaired consciousness. Finally, all emerging techniques for restoration of consciousness (for example, transcranial direct current stimulation, low-intensity focused ultrasound pulse, vagus nerve stimulation) will need to be compared in order to determine which is the most effective treatment on the basis of neurophysiological mechanisms [7].

Finally, we did not assess the neurobehavioral response with appropriate scales such as CRS-r. Due to the explorative nature of this study, we specifically assessed the autonomic response via the analysis of the EDA levels. We believe that this tool is more direct in response to the immersive VR stimulus in a brief therapeutic session. At the same time, it is necessary to correlate the neurobehavioral response of the patients to a course of treatment or longer sessions of VR intervention in subsequent studies.

### Implications of the Use of Immersive Virtual Reality in Clinical Practice

Immersive VR has been proposed as an effective intervention for motor and cognitive rehabilitation in several diseases. Although immersive systems in clinical practice require more evidence, our study supports naturalistic content in VR systems. In particular, naturalistic environments can induce the activation of the ANS, as demonstrated by changes in electrodermal activity. These modifications were independent of the scenario (static or dynamic). Our indications can support the use of these applications, offering multiple therapeutic strategies and guiding a tailor-made training program.

The immersive experience in VR is still far from being understood with regard to the emotional response elicited by the stimulating content. For several reasons, we argue for new longitudinal studies with larger samples (i) to test the effect of immersive VR in different clinical populations; (ii) to investigate which naturalistic (or personal) scenes are more likely to elicit positive outcomes; (iii) to analyze the value of using different immersive or non-immersive VR systems, avoiding potential negative outcomes or side effects; (iv) to examine the relevance of variation in physiological parameters as an evaluative marker for clinical practice; (v) to interpret the electrodermal response in relation to emotion in patients after severe acquired brain injury, as well as to perform a concomitant measurement of brain activity or brain imaging; and (vi) to measure the short-, medium-, and long-term effects of tailored immersive virtual reality guided by emotional responses.

## 5. Conclusions

The development of rehabilitation technology requires continuous adaptation and experimentation in different patient groups for extensive use. In particular, physical and/or cognitive impairments may limit the accessibility of therapeutic interventions. Based on the skin conductance response, our results show that adult individuals with impaired consciousness after an sABI are more activated after receiving naturalistic audiovisual content. In particular, immersive virtual reality stimulation elicited sympathetic arousal. These findings support the use of audiovisual VR stimulation to determine the clinical relevance in patients with severe clinical motor and cognitive conditions, such as patients with DoCs. A thorough understanding of the interaction between the ANS and DoCs is essential to provide comprehensive care and management to individuals with these disorders. This would also enable rehabilitation professionals to guide better treatment strategies and optimize patient outcomes, improving the overall well-being and quality of life of people with DoCs.

## Figures and Tables

**Table 1:** Demographic and clinical features of the enrolled patients at the enrolment in the experimental trial.

ID	Sex	Age (Years)	Etiology	DoC	CRS-r	GCS	LCF	Days from Acute Event
Patient 1	Male	69	Non-traumatic	MCS−	14	7	2	108
Patient 2	Male	22	Non-traumatic	UWS	9	7	2	173
Patient 3	Male	81	Non-traumatic	UWS	4	6	2	108
Patient 4	Male	29	Traumatic	MCS−	12	8	2	122
Patient 5	Male	52	Non-traumatic	MCS−	13	10	2	103
Patient 6	Female	66	Non-traumatic	MCS−	12	11	2	48
Patient 7	Female	70	Non-traumatic	MCS−	13	10	2	89
Patient 8	Male	21	Traumatic	MCS−	13	8	2	56
Patient 9	Female	66	Non-traumatic	UWS	4	6	2	24
Patient 10	Female	23	Traumatic	MCS+	16	6	3	50
Patient 11	Male	27	Traumatic	MCS+	18	12	3	35
Patient 12	Male	21	Traumatic	MCS+	16	11	3	26

DoC: Disorder of Consciousness; CRS-r: Coma Recovery Scale-revised; GCS: Glasgow Coma Scale; LCF: Level of Cognitive Functioning; UWS: Unresponsive Wakefulness Syndrome; MCS: Minimally Conscious State; MCS−: Minimally Conscious State Minus; MCS+: Minimally Conscious State Plus.

**Table 2:** The detailed scores for each item of Coma Recovery Scale-revised for the enrolled patients.

ID	Auditory	Visual	Motor	Oromotor/ Verbal	Communication	Arousal	Total Score
Patient 1	4	4	3	0	0	3	14
Patient 2	2	1	2	2	0	2	9
Patient 3	1	1	1	0	0	1	4
Patient 4	2	3	4	1	0	2	12
Patient 5	3	2	5	1	0	2	13
Patient 6	2	3	2	2	1	2	12
Patient 7	2	3	4	2	0	2	13
Patient 8	2	3	5	1	0	2	13
Patient 9	1	1	1	0	0	1	4
Patient 10	3	4	5	1	1	2	16
Patient 11	4	4	5	1	1	3	18
Patient 12	4	3	5	1	1	2	16

## Data Availability

The data presented in this study are available on request from the corresponding author.
